# Supplementary material for: Glo1 reduction in mice results in age- and sex-dependent metabolic dysfunction
Source: bioRxiv. 2025 Jan 25:2025.01.24.634754. Preprint. [Version 1] doi: 10.1101/2025.01.24.634754 (PMC11785252; doi:10.1101/2025.01.24.634754)
Supplement: Supplement 1 [file media-1.zip › Supplemental/Glo1 _Supplementary List.docx]

**Supplementary Materials List**

**Supplementary Table 1**

qPCR primer list

**Supplementary Figure 1**

Figure 1A: PCR genotype of WT and Glo1 mice at 3 weeks of age

Figure 1B: Glo1 enzyme activity at 28 weeks

**Supplementary Figure 2**

Figure 2A-2D: Food and water intake by WT and Glo1 mice

**Supplementary Figure 3**

Figure 3A: Schematic overview of Rage signaling

Figure 3B: Liver gene expression of Rage signaling

Figure 3C: Gonadal adipose gene expression of Rage signaling

Figure 3D: Kidney gene expression of rage signaling
