# Supplementary material for: Glo1 reduction in mice results in age- and sex-dependent metabolic dysfunction
Source: bioRxiv. 2025 Jan 25:2025.01.24.634754. Preprint. [Version 1] doi: 10.1101/2025.01.24.634754 (PMC11785252; doi:10.1101/2025.01.24.634754)

**Glo1 Supplementary Materials**

**Supplementary Table 1**

List of qPCR mouse primers

| **Gene** | **Forward primers** | **Reverse primers** |
| --- | --- | --- |
| *Glo1* | 5’-GCTTCTCCCACAAGTCTGTG-3’ | 5’-GGTACAGTGCAGGGGAAAGA-3’ |
| *Gapdh* | 5’-AACTTTGGCATTGTGGAAGG-3’ | 5’-ACACATTGGGGGTAGGAACA-3’ |
| *Rage* | 5′-AACACAGGAAGAACTGAAGCTTGG-3' | 5′-CTTTGCCATCGGGAATCAGAAGTT-3′ |
| *Akr1a1* | 5'-GGTATATTGTGCCCATGATTACG-3' | 5'-GGGGAGTAGCAGGCAATG-3' |
| *Aldh1a1* | 5'-GACAGGCTTTCCAGATTGGCTC-3' | 5'-AAGACTTTCCCACCATTGAGTGC-3' |
| *Lipin1* | 5'-CCCTCGATTTCAACGTACCC-3' | 5'-GCAGCCTGTGGCAATTCA-3' |
| *Acc1* | 5'-GGATATCGCATCACAATTGGC-3′ | 5′-CCTCGGAGTGCCGTGCTCTGGATC-3′ |
| *Fasn* | 5'-AGCGGCCATTTCCATTGCCC-3' | 5'-CCATGCCCAGAGGGTGGTTG -3' |
| *Elovl6* | 5’-CCCGAACTAGGTGACACGAT-3’ | 5’-TACTCAGCCTTCGTGGCTTT-3’ |
| *Scd1* | 5'-TTCTTGCGATACACTCTGGTGC-3' | 5'-CGGGATTGAATGTTCTTGTCGT-3' |
| *Srebp1c* | 5'-GGAGCCATGGATTGCACATT-3' | 5'-GGCCCGGGAAGTCACTGT-3' |
| *Dgat1* | 5'-GGAATATCCCCGTGCACAA-3' | 5'-CATTTGCTGCTGCCATGTC-3' |
| *Dgat2* | 5'-CCGCAAAGGCTTTGTGAA-3' | 5'-GGAATAAGTGGGAACCAGATCAG-3' |

**Supplementary Figure 1**

A.


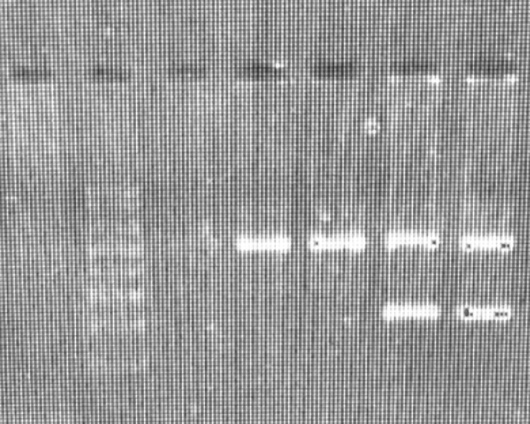


Gapdh (top band)

WT (no band)

Glo 1 KD (bottom band; 220bp)

B.

**Supplementary Figure 2**

**Supplementary Figure 3**


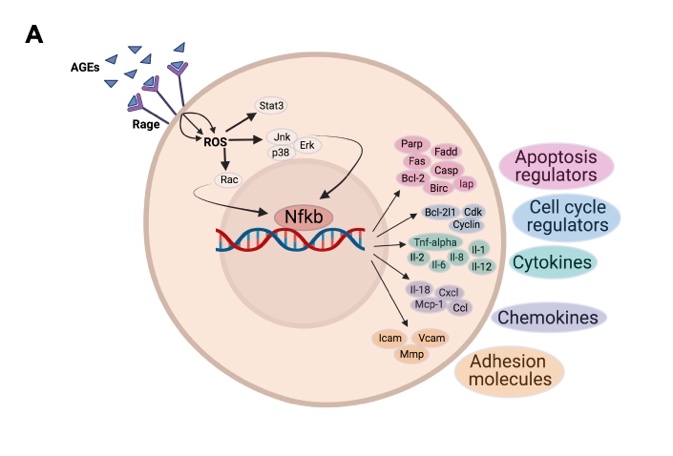

Supplement: Supplement 1 [file media-1.zip › Supplemental/Glo1_supplementary IC1.docx]
